# μ-2,5-Dihy­droxy­terephthalato-bis­[tri­aqua­(1,10-phenanthroline)zinc] dihy­droxy­terephthalate

**DOI:** 10.1107/S1600536812045837

**Published:** 2012-11-17

**Authors:** Hong Liu, Bo Liu, Ai-Ping Zhang, Chuan-Bi Li

**Affiliations:** a Department of Information & Technology, Jilin Normal University, Siping 136000, People’s Republic of China; b Department of Chemistry, Jilin Normal University, Siping 136000, People’s Republic of China

## Abstract

In the title compound, [Zn_2_(C_8_H_4_O_6_)(C_12_H_8_N_2_)_2_(H_2_O)_6_](C_8_H_4_O_6_), the complete ions of both the binuclear dication and the dianion are generated by crystallographic inversion symmetry. The Zn atom is bonded to an *N*,*N*′-bidentate phenanthroline ligand, three water moleules and an *O*-mono­denate 2,5-dihy­droxy­terephthalate dianion. In the resulting distorted octa­hedral ZnN_2_O_4_ coordination poly­hedron, the water O atoms are in a *mer* orientation. Two intra­molecular O—H⋯O hydrogen bonds occur in the bridging 2,5-dihy­droxy­terephthalate dianion within the complex cation and also in the free dianion. An intra­molecular O_w_—H⋯O (w = water) hydrogen bond also occurs within the dication. In the crystal, O—H⋯O hydrogen bonds link the component ions into a three-dimensional network.

## Related literature

For a related structure, see: Sun *et al.* (2007 ▶). For background to the applications of coordination polymers, see: Perry *et al.* (2009 ▶).
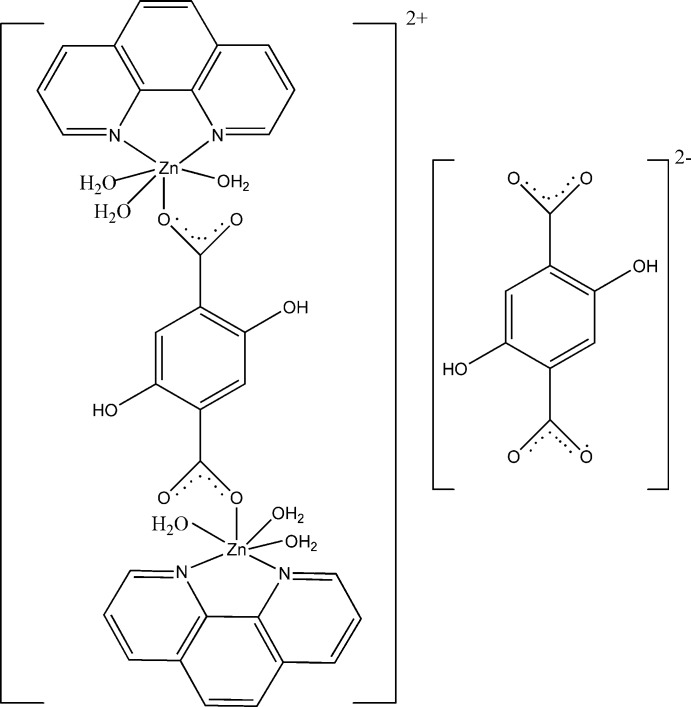


## Experimental

### 

#### Crystal data

[Zn_2_(C_8_H_4_O_6_)(C_12_H_8_N_2_)_2_(H_2_O)_6_](C_8_H_4_O_6_)
*M* *_r_* = 991.46
Triclinic, 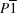

*a* = 8.765 (5) Å
*b* = 10.697 (5) Å
*c* = 11.062 (5) Å
α = 106.994 (5)°
β = 92.226 (5)°
γ = 90.977 (5)°
*V* = 990.7 (9) Å^3^
*Z* = 1
Mo *K*α radiation
μ = 1.30 mm^−1^
*T* = 293 K
0.25 × 0.18 × 0.15 mm

#### Data collection

Bruker SMART APEXII CCD diffractometer
Absorption correction: multi-scan (*SADABS*; Bruker, 2002 ▶) *T*_min_ = 0.737, *T*_max_ = 0.829
5446 measured reflections
3824 independent reflections
3205 reflections with *I* > 2σ(*I*)
*R*_int_ = 0.019

#### Refinement

*R*[*F* ^2^ > 2σ(*F* ^2^)] = 0.036
*wR*(*F* ^2^) = 0.086
*S* = 1.04
3824 reflections
289 parameters
H-atom parameters constrained
Δρ_max_ = 0.33 e Å^−3^
Δρ_min_ = −0.29 e Å^−3^

### 

Data collection: *APEX2* (Bruker, 2002 ▶); cell refinement: *SAINT* (Bruker, 2002 ▶); data reduction: *SAINT*; program(s) used to solve structure: *SHELXTL* (Sheldrick, 2008 ▶); program(s) used to refine structure: *SHELXL97* (Sheldrick, 2008 ▶); molecular graphics: *DIAMOND* (Brandenburg, 1999 ▶); software used to prepare material for publication: *SHELXTL*.

## Supplementary Material

Crystal structure: contains datablock(s) I, global. DOI: 10.1107/S1600536812045837/hb6960sup1.cif

Structure factors: contains datablock(s) I. DOI: 10.1107/S1600536812045837/hb6960Isup2.hkl

Additional supplementary materials:  crystallographic information; 3D view; checkCIF report

## Figures and Tables

**Table 1:** Selected bond lengths (Å)

Zn1—O1	2.0181 (19)
Zn1—O1*W*	2.184 (2)
Zn1—O2*W*	2.1581 (19)
Zn1—O3*W*	2.113 (2)
Zn1—N1	2.124 (2)
Zn1—N2	2.156 (2)

**Table 2:** Hydrogen-bond geometry (Å, °)

*D*—H⋯*A*	*D*—H	H⋯*A*	*D*⋯*A*	*D*—H⋯*A*
O1*W*—H1*WA*⋯O3^i^	0.97	1.95	2.902 (3)	170
O1*W*—H1*WB*⋯O2*W* ^ii^	0.92	2.01	2.911 (3)	168
O2*W*—H2*WA*⋯O2	0.93	1.75	2.663 (3)	166
O3—H3*A*⋯O2^iii^	0.82	1.84	2.562 (3)	147
O2*W*—H2*WB*⋯O5^iv^	0.91	1.80	2.692 (3)	166
O3*W*—H3*WA*⋯O4	0.89	1.85	2.695 (3)	158
O3*W*—H3*WB*⋯O4^iv^	0.83	1.82	2.650 (3)	175
O6—H6*A*⋯O5^v^	0.82	1.84	2.566 (3)	146

Symmetry codes: (i) 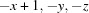; (ii) 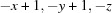; (iii) 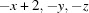; (iv) 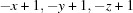; (v) 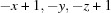.

**Fig. 1.**
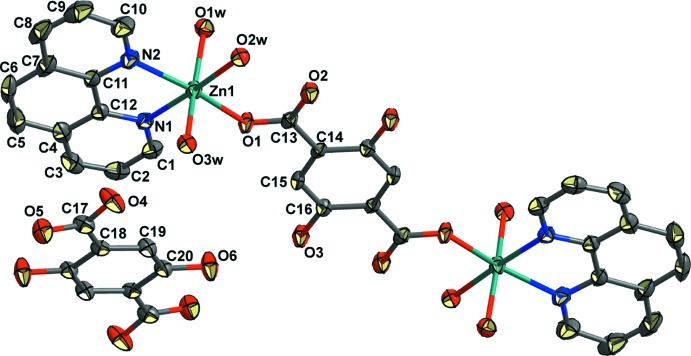
A representation of title compound. Displacement ellipsoids are drawn at the 40% probability level. H atoms have been omitted for clarity. Unlabelled atoms are related to the reference atoms by the symmetry operations. [Symmetry codes: (i) - x + 2, - y, - z; (ii) - x + 1, - y, - z + 1].

**Fig. 2.**
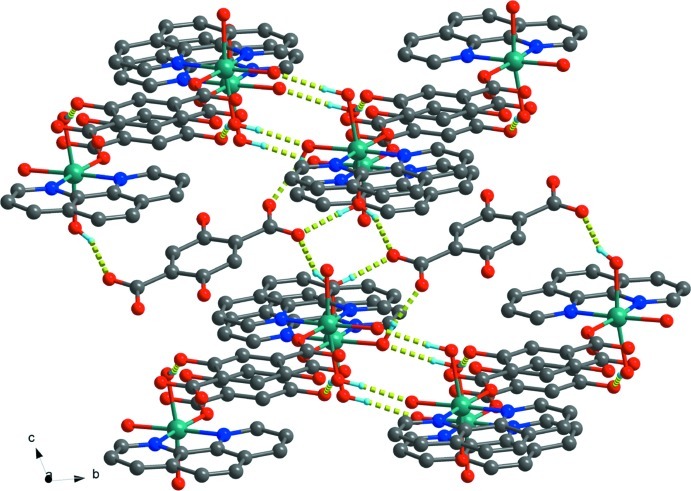
The packing diagram of the title compound. All H-atoms except for those involved in hydrogen bonds are omitted for clarity. (hydrogen bonds indicated by dashed lines).

## supplementary crystallographic information

### Comment

The design and synthesis of coordination compounds have attracted much interest in the fields of supramolecular chemistry and crystal engineering because of their intriguing structural diversities and potential applications (Sun *et al.*, 2007; Perry IV, *et al.*, 2009). To extend the previous work, we obtained the title compound, (I), by using Zn^II^, phenanthroline (phen) and 2,5-dihydroxyterephthalic acid (dhtp) as the starting materials.

The title compound, (I), is composed of a Zn^II^ canion, a phen molecule, half a coordinated dhtp anion, half a free dhtp anion and three coordinated water molecules in the asymmetric unit as shown in Fig. 1. Zn^II^ canion exhibits a distorted octahedral geometry, being coordinated by two N atoms of a phen molecule, one O atom from dhtp anion and three water O atoms. The Zn–O and Zn–N distances are normal. Zn^II^ canions are connected by dhtp anion to form a [Zn_2_(phen)_2_(dhtp)(H_2_O)_6_]^II^ cation unit. In additon, the free dhtp anion as the counter-ion presents in the sturcture. By way of O–H···O hydrogen bonding between the cation units and counter-anions, a three-dimensional network is formed (Fig. 2). The detailed hydrogen-bonding parameters are summarized in Table 1.

### Experimental

A mixture of Zn(CH_3_COO)_2_^.^2H_2_O (0.2 mmol), phen (0.3 mmol) and dhtp (0.2 mmol) were dissolved in 15 ml water. The resulting solution was stirred for about 0.5 h at room temperature, sealed in a 25-ml Teflon-lined stainless steel autoclave and heated at 443 K for three days under autogenous pressure. Afterward, the reaction system was slowly cooled to room temperature and colourless blocks of the title compound were recovered.

### Refinement

Carbon-bound H-atoms were positioned geometrically (C–H = 0.93 Å) and refined as riding, with *U*_iso_(H) fixed at 1.2*U*_eq_(C). Oxygen-bound for H3A and H6A atoms were positioned geometrically (O–H = 0.82 Å) and refined as riding, with *U*_iso_(H) fixed at 1.5*U*_eq_(O). In the case of coordinated water molecules, H atoms were clearly detected in a difference Fourier map, and refined freely. Final O–H bond length span the range 0.83–0.97 Å. Isotropic displacement parameters for H atoms were calculated as *U*_iso_(H) = 1.2*U*_eq_(C).

### Crystal data

**Table:**

[Zn_2_(C_8_H_4_O_6_)(C_12_H_8_N_2_)_2_(H_2_O)_6_](C_8_H_4_O_6_)	*Z* = 1
*M**_r_* = 991.46	*F*(000) = 508
Triclinic, *P*1	*D*_x_ = 1.662 Mg m^−^^3^
Hall symbol: -P 1	Mo *K*α radiation, λ = 0.71073 Å
*a* = 8.765 (5) Å	Cell parameters from 1867 reflections
*b* = 10.697 (5) Å	θ = 2.3–24.9°
*c* = 11.062 (5) Å	µ = 1.30 mm^−^^1^
α = 106.994 (5)°	*T* = 293 K
β = 92.226 (5)°	Block, colorless
γ = 90.977 (5)°	0.25 × 0.18 × 0.15 mm
*V* = 990.7 (9) Å^3^	

### Data collection

**Table:**

Bruker SMART APEXII CCD diffractometer	3824 independent reflections
Radiation source: fine-focus sealed tube	3205 reflections with *I* > 2σ(*I*)
Graphite monochromator	*R*_int_ = 0.019
ω scan	θ_max_ = 26.0°, θ_min_ = 1.9°
Absorption correction: multi-scan (*SADABS*; Bruker, 2002)	*h* = −10→5
*T*_min_ = 0.737, *T*_max_ = 0.829	*k* = −13→13
5446 measured reflections	*l* = −13→13

### Refinement

**Table:**

Refinement on *F*^2^	Primary atom site location: structure-invariant direct methods
Least-squares matrix: full	Secondary atom site location: difference Fourier map
*R*[*F*^2^ > 2σ(*F*^2^)] = 0.036	Hydrogen site location: inferred from neighbouring sites
*wR*(*F*^2^) = 0.086	H-atom parameters constrained
*S* = 1.04	*w* = 1/[σ^2^(*F*_o_^2^) + (0.0372*P*)^2^ + 0.484*P*] where *P* = (*F*_o_^2^ + 2*F*_c_^2^)/3
3824 reflections	(Δ/σ)_max_ = 0.001
289 parameters	Δρ_max_ = 0.33 e Å^−^^3^
0 restraints	Δρ_min_ = −0.29 e Å^−^^3^

### Special details

**Table:**

Geometry. All s.u.'s (except the s.u. in the dihedral angle between two l.s. planes) are estimated using the full covariance matrix. The cell s.u.'s are taken into account individually in the estimation of s.u.'s in distances, angles and torsion angles; correlations between s.u.'s in cell parameters are only used when they are defined by crystal symmetry. An approximate (isotropic) treatment of cell s.u.'s is used for estimating s.u.'s involving l.s. planes.
Refinement. Refinement of *F*^2^ against ALL reflections. The weighted *R*-factor *wR* and goodness of fit *S* are based on *F*^2^, conventional *R*-factors *R* are based on *F*, with *F* set to zero for negative *F*^2^. The threshold expression of *F*^2^ > 2σ(*F*^2^) is used only for calculating *R*-factors(gt) *etc*. and is not relevant to the choice of reflections for refinement. *R*-factors based on *F*^2^ are statistically about twice as large as those based on *F*, and *R*- factors based on ALL data will be even larger.

### Fractional atomic coordinates and isotropic or equivalent isotropic displacement parameters (Å^2^)

**Table:**

	*x*	*y*	*z*	*U*_iso_*/*U*_eq_	
Zn1	0.50422 (4)	0.32879 (3)	0.16460 (3)	0.03368 (11)	
C1	0.4116 (3)	0.0549 (3)	0.1904 (3)	0.0410 (7)	
H1	0.5020	0.0258	0.1515	0.049*	
C2	0.3222 (4)	−0.0322 (3)	0.2306 (3)	0.0490 (8)	
H2	0.3518	−0.1180	0.2177	0.059*	
C3	0.1916 (4)	0.0092 (3)	0.2888 (3)	0.0534 (9)	
H3	0.1321	−0.0478	0.3178	0.064*	
C4	0.1455 (3)	0.1379 (3)	0.3054 (3)	0.0468 (8)	
C5	0.0065 (4)	0.1877 (4)	0.3598 (3)	0.0634 (10)	
H5	−0.0560	0.1351	0.3920	0.076*	
C6	−0.0360 (4)	0.3089 (4)	0.3656 (3)	0.0643 (11)	
H6	−0.1282	0.3382	0.4008	0.077*	
C7	0.0574 (3)	0.3950 (3)	0.3186 (3)	0.0518 (8)	
C8	0.0161 (4)	0.5197 (4)	0.3177 (3)	0.0677 (11)	
H8	−0.0762	0.5528	0.3500	0.081*	
C9	0.1111 (5)	0.5934 (4)	0.2692 (4)	0.0735 (12)	
H9	0.0833	0.6761	0.2667	0.088*	
C10	0.2505 (4)	0.5432 (3)	0.2235 (3)	0.0600 (9)	
H10	0.3157	0.5949	0.1925	0.072*	
C11	0.1974 (3)	0.3504 (3)	0.2684 (3)	0.0386 (6)	
C12	0.2414 (3)	0.2194 (3)	0.2602 (2)	0.0357 (6)	
C13	0.7999 (3)	0.2201 (3)	0.0587 (2)	0.0319 (6)	
C14	0.9054 (3)	0.1076 (2)	0.0300 (2)	0.0274 (5)	
C15	0.8650 (3)	−0.0063 (2)	0.0586 (2)	0.0300 (6)	
H15	0.7743	−0.0104	0.0985	0.036*	
C16	0.9569 (3)	−0.1134 (2)	0.0290 (2)	0.0296 (5)	
C17	0.4052 (3)	0.2683 (3)	0.5940 (3)	0.0377 (6)	
C18	0.4543 (3)	0.1290 (2)	0.5460 (2)	0.0307 (6)	
C19	0.5797 (3)	0.0989 (2)	0.4716 (2)	0.0338 (6)	
H19	0.6340	0.1660	0.4531	0.041*	
C20	0.6264 (3)	−0.0285 (2)	0.4243 (2)	0.0334 (6)	
N1	0.3740 (2)	0.1776 (2)	0.2049 (2)	0.0346 (5)	
N2	0.2937 (3)	0.4250 (2)	0.2225 (2)	0.0419 (6)	
O1	0.6766 (2)	0.20561 (17)	0.10775 (18)	0.0382 (4)	
O2	0.8398 (2)	0.32217 (18)	0.0314 (2)	0.0447 (5)	
O3	0.9102 (2)	−0.22265 (18)	0.0592 (2)	0.0461 (5)	
H3A	0.9735	−0.2795	0.0370	0.069*	
O4	0.4677 (3)	0.35176 (18)	0.5525 (2)	0.0592 (7)	
O5	0.3055 (3)	0.29470 (18)	0.6758 (2)	0.0500 (5)	
O6	0.7485 (3)	−0.05155 (19)	0.3502 (2)	0.0563 (6)	
H6A	0.7647	−0.1301	0.3282	0.084*	
O1W	0.4148 (2)	0.28676 (19)	−0.03067 (18)	0.0434 (5)	
H1WA	0.3083	0.2617	−0.0317	0.052*	
H1WB	0.4161	0.3598	−0.0582	0.052*	
O2W	0.6253 (2)	0.49092 (17)	0.12905 (17)	0.0371 (4)	
H2WA	0.7050	0.4415	0.0879	0.045*	
H2WB	0.6629	0.5567	0.1971	0.045*	
O3W	0.5968 (2)	0.39846 (17)	0.35216 (17)	0.0380 (4)	
H3WA	0.5567	0.3612	0.4060	0.046*	
H3WB	0.5806	0.4777	0.3793	0.046*	

### Atomic displacement parameters (Å^2^)

**Table:**

	*U*^11^	*U*^22^	*U*^33^	*U*^12^	*U*^13^	*U*^23^
Zn1	0.03234 (18)	0.02944 (18)	0.04078 (19)	0.00700 (12)	0.01051 (13)	0.01119 (13)
C1	0.0402 (16)	0.0353 (15)	0.0467 (17)	−0.0007 (13)	−0.0064 (13)	0.0120 (13)
C2	0.058 (2)	0.0409 (17)	0.0477 (18)	−0.0125 (15)	−0.0175 (16)	0.0160 (14)
C3	0.061 (2)	0.057 (2)	0.0446 (18)	−0.0277 (18)	−0.0106 (16)	0.0206 (16)
C4	0.0400 (17)	0.063 (2)	0.0343 (16)	−0.0155 (15)	−0.0036 (13)	0.0107 (14)
C5	0.044 (2)	0.091 (3)	0.050 (2)	−0.018 (2)	0.0094 (16)	0.011 (2)
C6	0.0291 (17)	0.102 (3)	0.048 (2)	−0.0024 (19)	0.0123 (15)	0.000 (2)
C7	0.0350 (16)	0.069 (2)	0.0410 (17)	0.0148 (16)	−0.0012 (13)	−0.0009 (15)
C8	0.047 (2)	0.085 (3)	0.057 (2)	0.029 (2)	0.0002 (17)	−0.002 (2)
C9	0.081 (3)	0.058 (2)	0.074 (3)	0.040 (2)	−0.002 (2)	0.007 (2)
C10	0.068 (2)	0.050 (2)	0.065 (2)	0.0224 (17)	0.0092 (18)	0.0192 (17)
C11	0.0307 (14)	0.0508 (17)	0.0319 (14)	0.0062 (13)	0.0012 (12)	0.0081 (13)
C12	0.0293 (14)	0.0456 (16)	0.0305 (14)	−0.0035 (12)	−0.0027 (11)	0.0093 (12)
C13	0.0296 (14)	0.0303 (14)	0.0331 (14)	0.0045 (11)	0.0008 (11)	0.0048 (11)
C14	0.0230 (12)	0.0291 (13)	0.0270 (12)	0.0053 (10)	0.0003 (10)	0.0033 (10)
C15	0.0215 (13)	0.0336 (14)	0.0340 (14)	0.0037 (10)	0.0072 (10)	0.0076 (11)
C16	0.0285 (13)	0.0277 (13)	0.0316 (13)	−0.0013 (11)	0.0006 (11)	0.0074 (11)
C17	0.0541 (18)	0.0248 (14)	0.0333 (14)	0.0011 (12)	0.0027 (13)	0.0068 (11)
C18	0.0399 (15)	0.0221 (12)	0.0296 (13)	0.0005 (11)	−0.0001 (11)	0.0071 (10)
C19	0.0412 (15)	0.0210 (12)	0.0387 (15)	−0.0053 (11)	0.0038 (12)	0.0081 (11)
C20	0.0379 (15)	0.0276 (13)	0.0349 (14)	0.0012 (11)	0.0051 (12)	0.0091 (11)
N1	0.0306 (12)	0.0355 (13)	0.0381 (12)	0.0005 (10)	0.0001 (10)	0.0115 (10)
N2	0.0410 (14)	0.0395 (13)	0.0450 (14)	0.0138 (11)	0.0050 (11)	0.0111 (11)
O1	0.0308 (10)	0.0313 (10)	0.0540 (12)	0.0106 (8)	0.0151 (9)	0.0124 (9)
O2	0.0440 (12)	0.0337 (11)	0.0599 (13)	0.0102 (9)	0.0169 (10)	0.0167 (10)
O3	0.0384 (11)	0.0346 (11)	0.0705 (14)	0.0081 (9)	0.0190 (10)	0.0211 (10)
O4	0.1041 (19)	0.0225 (10)	0.0548 (13)	0.0075 (11)	0.0337 (13)	0.0132 (9)
O5	0.0615 (14)	0.0293 (10)	0.0564 (13)	0.0071 (10)	0.0205 (11)	0.0058 (9)
O6	0.0590 (14)	0.0323 (11)	0.0774 (16)	0.0039 (10)	0.0363 (12)	0.0113 (11)
O1W	0.0437 (12)	0.0450 (11)	0.0452 (11)	0.0008 (9)	0.0007 (9)	0.0191 (9)
O2W	0.0425 (11)	0.0287 (10)	0.0407 (11)	0.0044 (8)	0.0081 (9)	0.0097 (8)
O3W	0.0471 (11)	0.0270 (9)	0.0401 (11)	0.0057 (8)	0.0097 (9)	0.0090 (8)

### Geometric parameters (Å, º)

**Table:**

Zn1—O1	2.0181 (19)	C11—C12	1.437 (4)
Zn1—O1W	2.184 (2)	C12—N1	1.356 (3)
Zn1—O2W	2.1581 (19)	C13—O1	1.255 (3)
Zn1—O3W	2.113 (2)	C13—O2	1.263 (3)
Zn1—N1	2.124 (2)	C13—C14	1.497 (3)
Zn1—N2	2.156 (2)	C14—C15	1.389 (4)
C1—N1	1.324 (3)	C14—C16^i^	1.402 (3)
C1—C2	1.388 (4)	C15—C16	1.380 (3)
C1—H1	0.9300	C15—H15	0.9300
C2—C3	1.353 (5)	C16—O3	1.367 (3)
C2—H2	0.9300	C16—C14^i^	1.402 (3)
C3—C4	1.403 (5)	C17—O4	1.244 (3)
C3—H3	0.9300	C17—O5	1.258 (3)
C4—C12	1.407 (4)	C17—C18	1.505 (4)
C4—C5	1.422 (5)	C18—C19	1.384 (4)
C5—C6	1.339 (5)	C18—C20^ii^	1.401 (3)
C5—H5	0.9300	C19—C20	1.385 (4)
C6—C7	1.441 (5)	C19—H19	0.9300
C6—H6	0.9300	C20—O6	1.356 (3)
C7—C8	1.391 (5)	C20—C18^ii^	1.401 (3)
C7—C11	1.400 (4)	O3—H3A	0.8200
C8—C9	1.364 (6)	O6—H6A	0.8200
C8—H8	0.9300	O1W—H1WA	0.9650
C9—C10	1.397 (5)	O1W—H1WB	0.9182
C9—H9	0.9300	O2W—H2WA	0.9333
C10—N2	1.322 (4)	O2W—H2WB	0.9127
C10—H10	0.9300	O3W—H3WA	0.8873
C11—N2	1.362 (4)	O3W—H3WB	0.8287
			
O1—Zn1—O3W	93.06 (8)	C7—C11—C12	119.7 (3)
O1—Zn1—N1	90.52 (9)	N1—C12—C4	122.2 (3)
O3W—Zn1—N1	92.67 (8)	N1—C12—C11	117.9 (2)
O1—Zn1—N2	168.50 (8)	C4—C12—C11	119.9 (3)
O3W—Zn1—N2	90.35 (8)	O1—C13—O2	124.5 (2)
N1—Zn1—N2	78.34 (9)	O1—C13—C14	117.3 (2)
O1—Zn1—O2W	93.13 (8)	O2—C13—C14	118.2 (2)
O3W—Zn1—O2W	86.71 (7)	C15—C14—C16^i^	119.1 (2)
N1—Zn1—O2W	176.32 (8)	C15—C14—C13	119.7 (2)
N2—Zn1—O2W	98.03 (9)	C16^i^—C14—C13	121.2 (2)
O1—Zn1—O1W	90.61 (8)	C16—C15—C14	121.1 (2)
O3W—Zn1—O1W	171.35 (7)	C16—C15—H15	119.4
N1—Zn1—O1W	95.13 (8)	C14—C15—H15	119.4
N2—Zn1—O1W	87.58 (9)	O3—C16—C15	118.2 (2)
O2W—Zn1—O1W	85.27 (7)	O3—C16—C14^i^	122.0 (2)
N1—C1—C2	122.9 (3)	C15—C16—C14^i^	119.8 (2)
N1—C1—H1	118.6	O4—C17—O5	123.5 (3)
C2—C1—H1	118.6	O4—C17—C18	118.2 (3)
C3—C2—C1	119.2 (3)	O5—C17—C18	118.3 (2)
C3—C2—H2	120.4	C19—C18—C20^ii^	119.3 (2)
C1—C2—H2	120.4	C19—C18—C17	120.2 (2)
C2—C3—C4	120.3 (3)	C20^ii^—C18—C17	120.5 (2)
C2—C3—H3	119.9	C18—C19—C20	121.6 (2)
C4—C3—H3	119.9	C18—C19—H19	119.2
C3—C4—C12	117.0 (3)	C20—C19—H19	119.2
C3—C4—C5	124.0 (3)	O6—C20—C19	118.7 (2)
C12—C4—C5	119.0 (3)	O6—C20—C18^ii^	122.3 (2)
C6—C5—C4	121.2 (3)	C19—C20—C18^ii^	119.1 (2)
C6—C5—H5	119.4	C1—N1—C12	118.5 (2)
C4—C5—H5	119.4	C1—N1—Zn1	128.2 (2)
C5—C6—C7	121.7 (3)	C12—N1—Zn1	113.14 (18)
C5—C6—H6	119.1	C10—N2—C11	118.1 (3)
C7—C6—H6	119.1	C10—N2—Zn1	129.8 (2)
C8—C7—C11	117.5 (3)	C11—N2—Zn1	112.08 (18)
C8—C7—C6	124.1 (3)	C13—O1—Zn1	131.20 (17)
C11—C7—C6	118.4 (3)	C16—O3—H3A	109.5
C9—C8—C7	119.9 (3)	C20—O6—H6A	109.5
C9—C8—H8	120.1	Zn1—O1W—H1WA	107.0
C7—C8—H8	120.1	Zn1—O1W—H1WB	112.0
C8—C9—C10	119.3 (3)	H1WA—O1W—H1WB	105.5
C8—C9—H9	120.4	Zn1—O2W—H2WA	95.5
C10—C9—H9	120.4	Zn1—O2W—H2WB	118.0
N2—C10—C9	122.6 (4)	H2WA—O2W—H2WB	110.3
N2—C10—H10	118.7	Zn1—O3W—H3WA	114.7
C9—C10—H10	118.7	Zn1—O3W—H3WB	108.4
N2—C11—C7	122.6 (3)	H3WA—O3W—H3WB	106.6
N2—C11—C12	117.6 (2)		

Symmetry codes: (i) −*x*+2, −*y*, −*z*; (ii) −*x*+1, −*y*, −*z*+1.

### Hydrogen-bond geometry (Å, º)

**Table:**

*D*—H···*A*	*D*—H	H···*A*	*D*···*A*	*D*—H···*A*
O1*W*—H1*WA*···O3^iii^	0.97	1.95	2.902 (3)	170
O1*W*—H1*WB*···O2*W*^iv^	0.92	2.01	2.911 (3)	168
O2*W*—H2*WA*···O2	0.93	1.75	2.663 (3)	166
O3—H3*A*···O2^i^	0.82	1.84	2.562 (3)	147
O2*W*—H2*WB*···O5^v^	0.91	1.80	2.692 (3)	166
O3*W*—H3*WA*···O4	0.89	1.85	2.695 (3)	158
O3*W*—H3*WB*···O4^v^	0.83	1.82	2.650 (3)	175
O6—H6*A*···O5^ii^	0.82	1.84	2.566 (3)	146

Symmetry codes: (i) −*x*+2, −*y*, −*z*; (ii) −*x*+1, −*y*, −*z*+1; (iii) −*x*+1, −*y*, −*z*; (iv) −*x*+1, −*y*+1, −*z*; (v) −*x*+1, −*y*+1, −*z*+1.
